# Building a functional national One Health platform: the case of Tanzania

**DOI:** 10.1186/s42522-019-0003-0

**Published:** 2019-11-27

**Authors:** Andrew Y. Kitua, Susan Scribner, Mark Rasmuson, Dominic Kambarage, Janneth Mghamba, Elibariki R. Mwakapeje, Harrison Chinyuka, Jubilate Bernard, Kate Zimmerman, Sambe Duale, David Mutonga

**Affiliations:** 1 DAI Global Health / USAID/EPT-2 Preparedness and Response Project, Kampala, Uganda; 2 Public Health and Environment Advancement Interventions NGO ‘NGALAKERI’, Kihonda, Plot 980/81, White House, P.O. Box 5465, Morogoro, United Republic of Tanzania; 3 Mwalimu Julius Nyerere University, Musoma, United Republic of Tanzania; 4 Sokoine University of Agriculture (SUA), Morogoro, United Republic of Tanzania; 5 Ministry of Health, Community Development, Gender, Elderly and Children (MoHCDGEC), Dar-es-Salaam, United Republic of Tanzania; 6 Prime Minister’s Office, Disaster Management Department, Dar-es-Salaam, United Republic of Tanzania

**Keywords:** One health approach, Multisectoral coordination mechanism, Institutionalization, Tanzania

## Abstract

**Background:**

The USAID Preparedness and Response (P&R) project’s publication on Multisectoral Coordination that Works identified five dimensions most critical to creating effective and sustainable One Health platforms: political commitment, institutional structure, management and coordination capacity, technical and financial resources, and joint planning and implementation. This case study describes Tanzania experience in using these dimensions to establish a functional One Health platform. The main objective of this case study was to document the process of institutionalizing the One Health approach in Tanzania.

**Methods:**

An analysis of the process used to establish and institutionalize the MCM in Tanzania through addressing the five dimensions mentioned above was conducted between August 2018 and January 2019. Progress activity reports, annual reports and minutes of meetings and consultations regarding the establishment of the Tanzania national One Health platform were examined. Relevant One Health publications were studied as reference material.

**Results:**

This case study illustrates the time and level of effort required of multiple partners to build a functional multi-sectoral coordinating mechanism (MCM). Key facilitating factors were identified and the importance of involving policy and decision makers at all stages of the process to facilitate policy decisions and the institutionalization process was underscored. The need for molding the implementation process using lessons learnt along the way -- “sailing the ship as it was being built” -- is demonstrated.

**Conclusions:**

Tanzania now has a functioning and institutionalized MCM with a sound institutional structure and capacity to prevent, detect early and respond to health events. The path to its establishment required the patient commitment of a core group of One Health champions and stakeholders along the way to examine carefully and iteratively how best to structure productive multisectoral coordination in the country. The five dimensions identified by the Preparedness and Response project may provide useful guidance to other countries working to establish functional MCM.

## Background

The USAID Preparedness and Response (P&R) Project, part of USAID’s Emerging Pandemic Threats-2 program (EPT-2), supported the development and strengthening of One Health (OH) multisectoral coordination mechanisms or platforms in 16 countries in East Africa, West Africa, and Southeast Asia over a period of 4 years (2014–2018). A research study conducted by the project (*Multisectoral Coordination that Works)* identified five dimensions most critical to effective and sustainable One Health platforms [1]:

- *Political Commitment*: A legal mandate is essential to establish a formal multisectoral coordination mechanism and help it survive political changes. Continuous advocacy is needed to help national decision-makers to understand the mechanism’s value in prevention, detection, and response.
- *Institutional Structures*: A formal structure helps ensure mechanisms are functional, effective, and sustainable. Clarity in organization and terms of reference is central to secure government ownership, build stakeholder engagement, and develop capacity to prepare and respond to a public health event.
- *Management and Coordination Capacity*: Coordination requires good management and leadership “soft skills”. Management and coordination capacity at all levels are critical, while annual work plans, communications guidelines, and monitoring and evaluation frameworks support it.
- *Technical and Financial Resources*: Sustained multisectoral coordination requires national government ownership, leadership, and resources. National governments can also mobilize and coordinate investments from development partners, research institutions, and the private sector.
- *Joint Planning and Implementation*: Builds relationships and strengthens trust among partners. It also demonstrates the value of multisectoral coordination, as resulting plans and activity reports document the benefits gained.

This case study describes how Tanzania built and institutionalized its national OH platform through paying attention to all the five dimensions and was able to perform several key activities to advance preparedness and response and health security along the way.

## Methods

An analysis of the process used to establish and institutionalize the MCM in Tanzania and through addressing the five dimensions mentioned above was conducted between August 2018 and January 2019. Progress activity reports, annual reports and minutes of meetings and consultations regarding the establishment of the Tanzania national One Health platform were examined. Relevant One Health publications were studies as reference material. The main objective of this case study was to document the process of institutionalizing the One Health approach in Tanzania.

## Results

### Generating political commitment

Shocks lead to action. The Tanzanian resolve to adopt the One Health (OH) approach came about after the Rift Valley Fever (RVF) outbreak of 2006–2007 [2]. The devastating effects of RVF on human and animal health, as well as on the economy [3, 4], prompted the Prime Minister’s Office (PMO) to take on the coordination and leadership role of the response, which was implemented jointly by the ministry responsible for public health and social welfare and the ministry responsible for livestock. After the end of the outbreak, these coordination efforts lost momentum, but this and previous epidemics had catalyzed positive reaction at academic and research institutions, towards strengthening national systems to address health threats with better coordination (Table 1).

**Table 1:** Facilitating factors for the adoption of the One Health approach in Tanzania.

1	Sock of the Rift Valley Fever 2006-2007
2	Policy decision by the East African Sectoral Council of Ministers of health, April 2014
3	Motivated One Health networks initiatives championed by Universities and Research institutions
4	Technical advice and support provided by the P&R project

Building upon the East African Integrated Disease Surveillance Network, a tripartite partnership between academia, research groups and the government established in 2000 [5], Tanzanian institutions joined and became active members of a number of regional One Health networks which were being created including the Cysticercosis Working Group in East and Southern Africa (CWGESA) in 2002; African Field Epidemiology Network (AFENET) in 2005; the Southern African Centre for Infectious Disease Surveillance (SACIDS) in 2008; Afrique One-ASPIRE in 2009; One Health Central and East Africa (OHCEA) in 2010; and; Global Anti-Microbial Resistance Partnership-Tanzania (GARP-TZ) in 2012.

In 2013 institutions championing OH in Tanzania felt the lack of cohesiveness among their networks and launched an initiative that would officially pull together institutions and One Health networks to harmonize their activities with the aim of minimizing duplication and maximizing efforts to fight communicable diseases. This was viewed as a roadmap towards the development of an agenda prioritizing OH activities. The initiative strengthened engagement and collaboration between the above-mentioned networks and government sector ministries dealing with human health, animal health (livestock and wildlife), and the environment. It recognized the need for greater multisectoral and multidisciplinary coordination and development of a national One Health Agenda. It was launched in Arusha in February 2013 by the Tanzanian vice president.

In October 2015, responding to the policy statement of the East African Sectoral Council of Ministers of Health issued April 2014 urging partner states to adopt a OH approach in their country policies [6], Tanzania developed a national One Health Strategic Plan for 2015–2020. This milestone strengthened dialogue about strengthening coordination among the above-mentioned networks, leading to proposals for a national One Health Forum. A stakeholders’ meeting was organized from 10 to 14 February 2016 to discuss and agree on the structure and functions of the proposed Forum. The USAID Preparedness and Response Project (P&R) had begun working in East Africa in early 2015, establishing a regional office in Kampala, and the project’s Regional OH Technical Advisor, who was in discussions with Tanzania government officials about the initiation of the P&R project and its objectives, attended the above mentioned stakeholders’ meeting.

The meeting agreed with the idea promoted by P&R Project of establishing a multisectoral OH coordinating mechanism or National One Health Platform (NOHP) under the PMO’s office, rather than a Forum. This would better serve institutionalization of the OH approach within government’s structure as urged by the EA Council of Ministers in 2014. This would further allow budget allocation for the coordination office since it would be an organ of the national Disaster Management Department (DMD) and One Health activities would access funds budgeted for disaster management. In 2016, P&R Project appointed a National One Health Technical Advisor to assist the country in establishing the OH coordination mechanism and institutionalizing One Health.

#### Early challenges

In establishing a OH coordination structure in Tanzania, several challenges appeared, including operationalization of the already developed National OH Strategic Plan. A stakeholders meeting was convened in Dar es Salaam on August 22–23, 2016, to discuss operationalization of the Strategic Plan, including the state of the national One Health Coordinating Unit (OHCU). The meeting concluded that there had been inadequate stakeholders’ consultations in outlining the structure of the OHCU, its point of linkage to the PMO, and how it would access resources including finance and staff, to enable it to be operational. Participants observed several operational deficiencies including: (1) naming the One Health coordinating mechanism a *Unit* would not fit with the government structure and positioning under the PMO; (2) seconding personnel to the Unit from sector ministries was not practical and not aligned with Government’s formal employment procedures and guidelines; and (3) the technical working groups proposed were too broad in scope and lacking in key expertise to be functional (Table 2).

**Table 2:** The essentials for operationalization of national plans

1	Good and well-intentioned plans may not be implemented if high level policy and decision makers are not involved at the early stages of their development
2	High level policy and decision makers need to be sensitized and well informed about the benefits of intended national plans
3	Their engagement at the very beginning is necessary to allow conformity with national regulations, systems and structures.
4	They hold the final decision on national resources allocation including budget allocations

The meeting recommended studying, understanding and aligning with the national employment policies and procedures; creation of a few strategic technical working groups (TWGs) composed of key technical OH champions; and consultations with the PMO for guidance on the way forward.

#### Advocacy and consensus-building

The above concerns and recommendations were further discussed in two stakeholders’ meetings held respectively in April and September 2017 respectively and coordinated by the OHCU, which was then constituted as an interim coordinating body working virtually without a formal office. The outcome was the appointment of a Task Force comprised of OH experts and champions to conduct face-to-face discussions with high-level policy and decision makers of key sector ministries and the Prime Minister’s Office. These consultations, resulted in recommendations for engaging policy and decision makers and key institutional representatives in all stages of OH planning and implementation; continuous sensitization and education of key policy and decision makers since many of those consulted had only a vague idea about One Health and why its institutionalization was so important; repeated advocacy over time to address high government staff turnover; and formal launching of the legally established coordination unit and the revised OH Strategic Plan.

### Creating a sound institutional structure

Responding to the advocacy efforts outlined above and acting on the recommendations of the Task Force, the PMO agreed to:

- Change the name of the OHCU to national One Health Coordination Desk (OHCD) allowing it to fit within the existing structures of the DMD.
- Employ full time staff for the OHCD.
- Nest the OHCD within the Disaster Management Department (DMD) of the PMO office.
- Institutionalize the OH approach at subnational levels, with coordination and leadership of the OHCD.

**Fig. 1:**
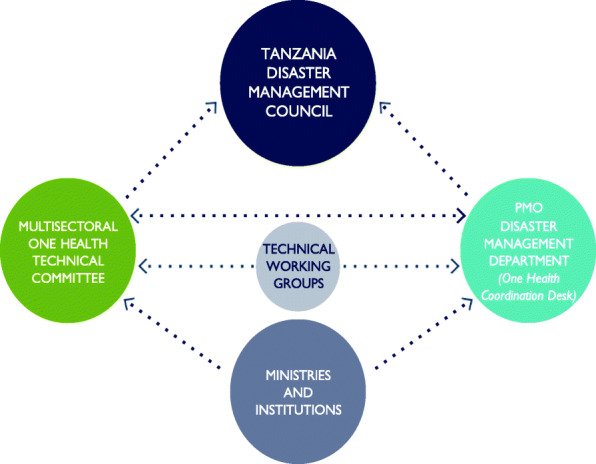
Tanzanian National One Health Platform

The revised structure provides for a platform comprised of the One Health Coordination Desk (OHCD) within the DMD, four thematic technical working groups (Training, Advocacy and Communication; Preparedness and Response; Research and Development; Surveillance), and a National Multisectoral One Health Technical Committee that brings together directors from core OH Sector ministries (human health, animal health (livestock and wildlife), and the environment) and agencies under the chairmanship of the Director of the DMD (See Fig. 1 below). The existing Tanzania Disaster Management Council chaired by the permanent secretary for policy and planning of the PMO will play the role of the steering committee. The President’s Office - Regional Administration and Local Governments (PO-RALG) was also incorporated into the revised structure to reflect sub-national OH coordination arrangements. The OHCD which was still operating with interim staff, will comprise of three full time staff employed by the PMO, including a medical epidemiologist/public health expert, a veterinary epidemiologist expert, and an ecologist or wildlife expert. Additional experts may be coopted to support specific functions of any of the committees as need may arise. Separate sector and agency focal persons will act as the direct link to their ministries.

There is a legal binding document concerning inter-institutional and inter-sectoral communication, collaboration, and coordination on a routine basis and during a response to various public health emergencies in the form of a memorandum of understanding (MoU). The National One Health Strategic Plan 2015–2020 (NOHSP) stipulates tasks for each institution, and the One Health for Central and East Africa (OHCEA) platform oversees long-term collaboration among the academic institutions in the region. The NOHSP envisions a nation with optimal health for people, animals and the environment achieved through collaborative efforts locally, nationally, regionally and globally. Its mission states: Improve the well-being of the United Republic of Tanzania by promoting collaboration in addressing One Health country priorities. Its goals are:

1. Increase awareness on One Health for professionals, policymakers and the community
2. Strengthen preparedness planning and improve the ability to respond to zoonotic disease outbreak at all levels (community, District, Regional and National)
3. Improve the health of humans, animals and the environment through evidence-based research
4. Provide functional and quality integrated human and animal health systems, at all levels, to reduce the burden of zoonotic diseases.
5. Strengthen institutional frameworks to support One Health implementation

Each goal has strategic objectives and timelines as summarized in Table 3 below.

**Table 3:** Goals, Strategic Interventions and Objectives of the Tanzania National One Health Strategic Plan

Goals	Strategic Objectives	Timeline
Increase awareness on One Health for professionals, policymakers and the community	To increase awareness about One Health to 80% of at-risk population from identified priority zoonotic disease areas at all levels.	June 2020
	To increase the knowledge base focused on One Health initiatives in 80% of preservice and 30% of in-services trainings.	July 2018
	To increase awareness about One Health to 100% of policy and decision makers.	July 2020
Strengthen preparedness planning and improve the ability to respond to zoonotic disease outbreak at all levels (community, District, Regional and National)	To enhance institutional collaborative research projects on One Health by 60%.	2020
	To coordinate the setting of One Health research priorities among all institutions.	2020
	To Enhance communication linkages between One Health researchers and policy Makers.	2020
Improve the health of human, animal and environment through evidence-based research	To enhance institutional collaborative research projects on One Health by 60%.	2020
	To coordinate the setting of One Health research priorities among all institutions.	2020
	To Enhance communication linkages between One Health researchers and policy Makers.	2020
Provide functional and quality integrated human and animal health systems, at all levels, to reduce the burden of zoonotic diseases	To promote and strengthen integrated surveillance, prevention and control of zoonotic diseases in 12% of the districts annually.	2020
	To enhance zoonotic disease diagnostic capacity at the national level (100%), zones and regions (60%) and district levels (30%).	2020
Strengthen institutional framework to support One Health implementation	To establish a cost effective and efficient One Health coordinating unit involving 80% of stakeholders, to be housed within the DMD PMO’s office.	July 2016
	To establish a mechanism to facilitate and collaborate One Health activities with the relevant ministries, agencies and other organizations by average of 60%.	2020

**Fig. 2:**
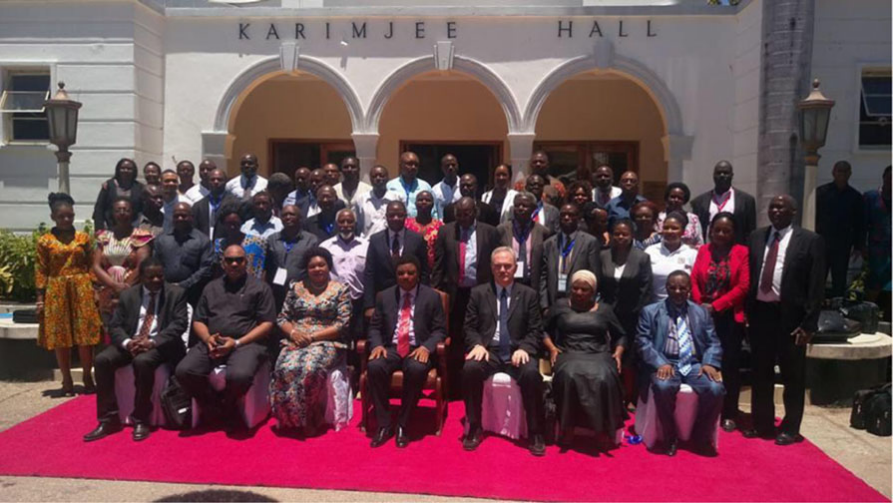
Launch of the OH strategic plan 1015-2020 and One Health Coordinating Desk in Dar es Salaam. Courtesy: Preparedness and Response Project, 2018

The One Health Coordination Desk and the Tanzanian National One Health Strategic Plan were launched on February 13, 2018, with firm government commitment to their operationalization [7]. The launch ceremony was presided over by the Hon. Kassim M. Majaliwa (MP), Prime Minister of the United Republic of Tanzania. In his remarks, the Hon. Prime Minister stated that “The United Republic of Tanzania is committed to achieving all the health security capacities, not only for Tanzania but also to contribute towards global health security. With the One Health Coordination Desk launched, the Government of Tanzania is committed to coordinated action and information sharing across health sectors to minimize outbreaks and save lives” (Fig. 2).

### Ensuring management and coordination capacity

There are multiple constituent parts to the One Health Platform, all of which require skillful coordination. The OHCD plays a central secretariat role, managing day-to-day coordination of OH activities, convening stakeholders for planning and sharing information, monitoring progress of implementation, reporting on activities, providing logistic support to the TWGs, and coordinating with various partners, including NGOs and universities.

The national multisectoral One Health Technical Committee (OHTC) brings together directors responsible for responding to health threats from core OH ministries and agencies under the chairmanship of the Director of the Disaster Management Department. Table 4 below shows the core OH sector ministries, institutions and partners represented on the One Health Technical Committee.

**Table 4:** Core ministries, institutions and partners represented on One Health Technical Committee

Prime Minister’s Office -Disaster Management Department (Secretariat)	
Ministry responsible for livestock and fisheries	
Ministry responsible for public health and social welfare	
Ministry responsible for tourism and natural resources (wildlife, Tanzania National Parks)	
Ministry responsible for environment	
Ministry responsible for agriculture, food security and cooperatives	
Ministry responsible for education and vocational training (universities)	
Ministry responsible for communication/broadcasting	
Commission for Science and Technology	
Development Partners Group of Tanzania	

The structures, processes, and resources for preparedness and response against health threats are within these ministries. As implementers of policies and practices, they therefore hold the key to successful collaboration entailing joint planning, mounting joint interventions and response, and sharing resources as well as information.

In addition to the participation of these ministries and partners, there are other partners who participate on the four Technical Working Groups (TWGs)—Surveillance, Preparedness and Response, Research and Development (R&D), and Training, Advocacy, and Communication (TAC). For example, the Ministry responsible for finance and planning affairs and Ministry responsible for home affairs (internal security and immigration) participate in the Preparedness and Response TWG; the Ministry responsible for defense and national services participates in both the Preparedness and Response and Surveillance TWGs; Tanzania Medical Association participates in the Preparedness and Response and Surveillance TWGs; various research institutions participate in the Surveillance, Preparedness and Response, and R&D TWGs; the National Environment Management Council (NEMC) and other relevant sectors (water, security, transport, immigration) also participate in the R&D TWG; the World Health Organization, Food and Agriculture Organization, and private sector all participate in the Preparedness and Response TWG.

**Fig. 3:**
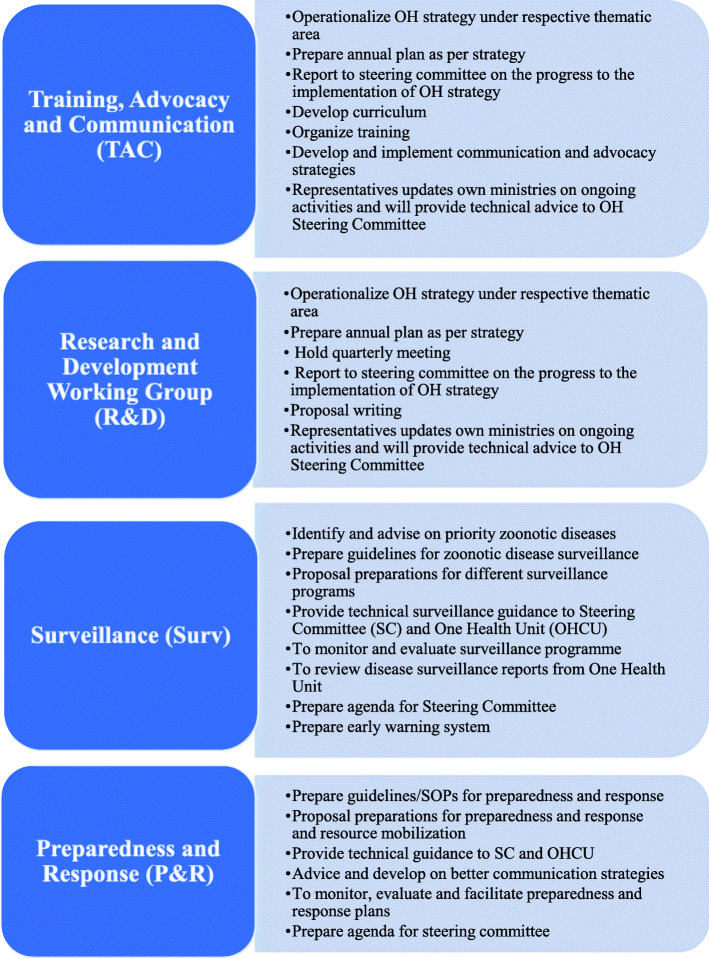
Technical Working Groups and their respective terms of references

The Terms of Reference (TOR) of the TWGs are listed in Fig. 3 below. A broad range of disciplines is represented among the four TWGs. For example, both the Surveillance TWG and Preparedness and Response TWGs include both human health and veterinary epidemiologists as well as laboratorians and social scientists. There is room for any TWGs to coopt temporarily any extra technical expertise on need. In order to strengthen its capacity to undertake the coordination role, the P&R project supported and facilitated a leadership training of the OHCD staff and OH TWGs, building skills in working across sectors, organizing effective meetings, and managing differences.

#### The critical role of the Prime Minister’s Office

The work of the OHCD is greatly strengthened and facilitated by its location within the Prime Minister’s Office, whose central role of coordinating multisectoral actions is provided by its mandate of approving and directing ministers’ actions. It stands in a position of no conflict of interest, able to arbitrate issues among the sectors since national security is vital and concerns all sectors. Positioning the OHCD under the PMO allows true ownership and leadership while providing optimal coordination of national and international efforts. The One Health platform is hence enabled to serve all sectors, partners and other stakeholders with no conflict of interest (Table 5).

**Table 5:** The rationale for establishing One Health coordination under the Prime Minister’s Office (PMO)

1	Everyone agrees to the need for coordination, but no one likes to be coordinated
2	PMO has the legal mandate to coordinate and direct sector ministries
3	It bears no conflict of interest in any single sector activities
4	It influences national resource allocation including budgeting

### Mobilizing technical and financial resources

Recognizing that the successful operationalization of its One Health strategic plan requires availability of adequate resources (infrastructure, human and finance), the Tanzanian One Health platform has developed a resource mobilization strategy capable of providing adequate resources for operationalizing the plan. This involved a series of planning and advocacy meetings with strong engagement of the Directorate of Policy and Planning (DPP) in the Disaster Management Department (DMD) of the Prime Minister’s Office, and the Ministry of Finance.

These meetings culminated in: (1) Tasking the DMD to develop and submit a proposal through PMO for a government funding allocation of Tanzanian shillings 600 million i the financial year 2018–2019 (DMD to make this a priority and fast track the submission); (2) Demanding sector ministries to mainstream collaborative activities into their annual budgets; (3) Requesting the development of an operational framework for the OHCD that enables transparency and accountability by tracking mobilization of resources, their availability and use; and for (4) empowering the OHCD to (a) mobilize resources through submission of fundable proposals to national financing sources and global financing facilities, (b) strengthen collaboration with the Directorate of Policy and Planning; and (c) engage academia and research experts to team up with the OHCD to write and submit fundable proposals; this will allow the OHCD to gain experience and learn from existing experts in academia and research.

A task force was organized by the OHCD to analyze the NOHSP to identify needed resources and available resources and suggest a plan of action for the development of the National One Health Resource Mobilization Strategy (NOHRMS). A consultant was hired by P&R Project to work in close collaboration with the task force and through a series of consultations develop a draft NOHRMS. The draft was reviewed at a OH stakeholders’ workshop in June–July 2018 in Dar es Salaam and subsequently submitted to DMD and validated in August 2018 (Table 6).

**Table 6:** Indicators of effective national ownership and leadership of OH Coordination

1	Strong commitment to national resource allocation
2	Commitment to resource sharing among the key sectors
3	Mapping of available resources
4	Development and implementation of a resource mobilization strategy

#### Goal and pillars of the NOHRMS

The overall goal is to mobilize financial, human and logistic resources that are essential for effective and sustainable implementation of the NOHSP. The strategy stands on three pillars:

1. Predictable and sustainable financial resources
2. Sharing of expertise and physical resources
3. Requisite capacity to mobilize and/or coordinate resources mobilization.

The first pillar recognizes that successful implementation of the NOHSP will depend largely on availability of predictable and sustainable resources especially from the government (Table 7). Accordingly, it calls for establishing a One Health program code and budget at the PMO by March 2019; mainstreaming One Health into sector budgets and strategies by 2020; integrating private sector and One Health consortia in One Health implementation; and establishing an accountability framework for PMO and One Health sectors over resources mobilized and activities implemented by 2020.

**Table 7:** The foundation of predictable and sustainable financial resources

1	Annual national budget allocation
2	Long term financial commitments
3	Capacity and skills to engage the government and external financial institutions

The second pillar--sharing of expertise and physical resources--recognizes that considerable resources for prevention, detection and response to health threats already exist in the key OH sectors, academic and research institutions, the private sector, OH Networks and projects. Access to and sharing of these would optimize use of limited resources and maximize impact. It targets achieving optimal sharing of resources (expertise, laboratory and other physical structures including transport) by 2020.

Strengthening the OHCD’s capacity to take leadership in coordinating resource mobilization among OH stakeholders--the third pillar--is critical for successful implementation of the strategic plan. The NOHRMS outlines specific actions to help OHCD staff acquire requisite skills and capacities to engage governments and donors to mobilize, and account for, resources by 2020. It includes appendices containing guidelines, concept notes and Standard Operating Procedures (SOPs) required for its successful implementation.

The NOHRMS validation meeting agreed on an operational framework detailing all activities to be implemented to achieve coordinated mobilization and sharing of resources (infrastructure, human and financial) for the successful implementation of the activities outlined in the NOHSP. It tasked the OHCD to lead the implementation of the strategy in close collaboration with line ministries and key One Health actors through the One Health focal points.

### Joint planning and implementation

Even as the Tanzanian government was building the structure of its One Health platform through the methodical process of consultation and consensus building described above, the platform was performing important new functions--“sailing the ship as it was being built”. Hence despite not yet securing regular government funding, the platform is already functional and has already taken several actions including the ones described below using current budget allocations and support from partners and projects. In doing so it reinforced the platform’s collaborative functions and value, as illustrated by the following examples showing the strength of unity (Table 8).

**Table 8:** The strength of joint action (Umoja ni nguvu!*)

1	Working in silo is limiting, as no single sector can have all the necessary resources to adequately address a health threat
2	Resource sharing is critical in resource limited settings to maximize efficiency and impact
3	Response to the anthrax outbreak of 2016 illustrated the strength of joint actions

*Kiswahili wisdom meaning “Unity is strength”

#### Anthrax outbreak response in 2016

Anthrax outbreaks are frequent in Tanzania and there is evidence of increasing incidence in the period 2013–2017 [8, 9]. Arusha and Kilimanjaro register higher incidence than other parts of Tanzania. Signals of an outbreak in late 2016 began as rumors of mass deaths of wild animals in Monduli District, Arusha Region, in late October and early November 2016 [9]. Monduli is one of the five districts in Arusha Region, located in northern Tanzania, within the Great East African Rift Valley. The district forms part of the northern tourist circuit surrounded by some of the world’s most famous natural wildlife attractions to visitors from around the world. These rumors were followed by reports of livestock deaths in middle November, and towards the end of November reports of human cases reached the Ministry of Health.

Initial reports indicated there were 19 suspected human cases, 31 livestock deaths (11 cattle, 17 goats and 3 sheep); and 103 wildlife animal deaths (89 wildebeests, 13 grand gazelles and one hare.)

The National One Health Coordinating Unit was tasked to coordinate the response. A central multisectoral response team was formed comprising Ministry of Health, Community Development, Gender, Elderly and Children (MoHCDGEC); Regional Medical Officer’s Office; and Tanzania Wildlife Research Institute (TAWIRI). It teamed up at the district level with the District Medical Officer (DMO); District Veterinary Officer (DVO); and District Game Office (DGO).

**Fig. 4:**
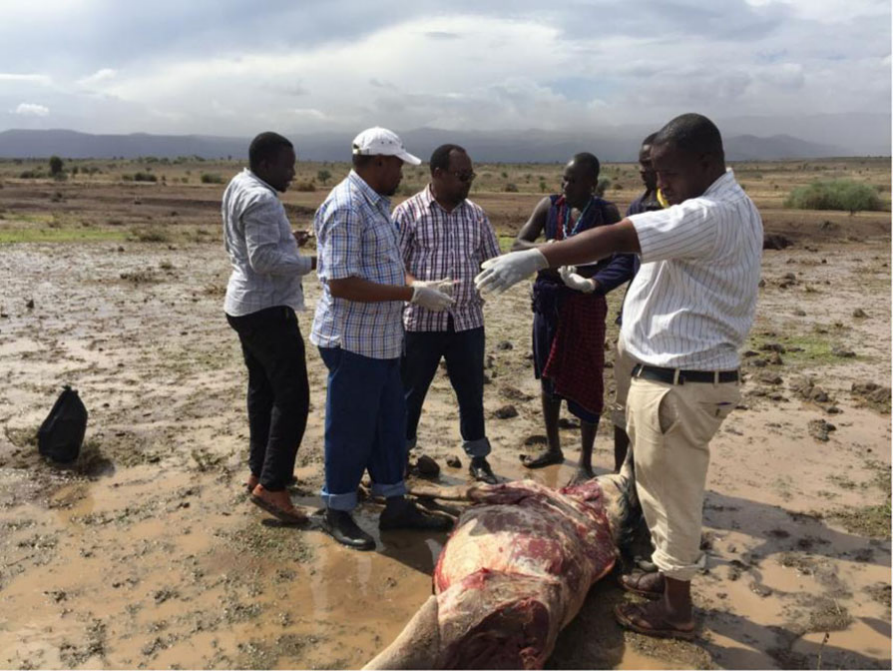
A carcass of a wildebeest in Monduli district, Arusha region, Tanzania (Courtesy: Elibariki M. 2016)

Through interviews with household members and observation, it was evident that households with reported cases of anthrax were close to where wildebeest carcasses were spotted, and that there was a history of consuming meat from the carcasses. Members of households acknowledged a long history of their domestic animals interacting with wild herbivores like wildebeest, zebra, and impala, and admitted to consuming meat from dead carcasses during grazing their animals (Fig. 4).

Interviewed livestock keepers said that they normally treat their sick animals by themselves and only consult livestock field officers when faced with complications. Dying animals are killed and their meat shared among household members and often with neighbors.

The field team visited sites with decomposing wildlife carcasses. Samples collected from these wildlife carcasses later tested positive for *Bacillus anthracis* at the Arusha Center -Tanzania Veterinary Laboratory Agency (TVLA). Examination and interviews of members of nearby “boma” (a collection of huts forming a family homestead) revealed the presence of skin lesions suggestive of cutaneous anthrax among members who testified to have consumed a dead animal’s meat and used the skin of wildlife as bedding material.

The outbreak investigation team concluded from the laboratory results and observations that anthrax in wildlife carcasses was the likely source of that outbreak. This finding was justified by the evidence of deaths of wildlife animals that tested positive for Bacillus anthracis and clinical manifestation of anthrax in human cases that consumed meat of wildlife cadavers.

The team recommended systematic risk assessment, risk communication and enhancing community sensitization.

Given the difficulty of controlling the disease in wildlife, the team also recommended reducing human and livestock interaction with wildlife to minimize the risk of infection spillover and spread to human populations.

#### After action review

An After-Action Review was coordinated by the NOHP in 2017. Again, a multisectoral team was assembled to conduct a review of the outbreak investigation through examination of records, reports and conducting interviews with local officials and community members of the affected region. The AAR concluded that the outbreak response was rapid and efficient and benefitted highly from its multisectoral composition, a statement supported by others [7]. It prevented the outbreak from spreading from source, saving lives and minimizing the economic loss to livestock and tourism sectors [7].

But the AAR also identified several preparedness and response gaps, including poor multisectoral coordination and information sharing at the sub-national levels; delayed detection, reporting and laboratory confirmation especially in the animal health sector; limited workforce, tools, supplies and logistics, which were more pronounced in the animal health sectors; and an uncoordinated animal vaccination system with very low coverage. It identified key Anthrax infection drivers being negative cultural beliefs and practices such as consuming meat from dead or sick animals and poor knowledge of the cause of Anthrax infection.

It recommended enhancement of community health education through a comprehensive risk communication strategy involving social scientists/anthropologists; integrating human and animal diseases surveillance data and information sharing among different sectors; review and updating of the national animal vaccination program; and strengthening multisectoral collaboration at all levels. The recommendations from the AAR meeting were incorporated into the national anthrax prevention and control strategy (2018–2023), which has been developed with the technical assistance from FAO.

Subsequently, the Arusha Region established its own regional One Health Team, and the Tanzania Veterinary Laboratory Agency (TVLA) is committed to work with the ministry responsible for livestock to revamp the animal vaccination program to address the deficiencies observed at the sub-national level.

#### Strengthening preparedness and response plans

The nascent National One Health Coordination Desk also undertook the revision of the Tanzanian Emergency Preparedness and Response Plan (TEPRP) in September 2017, with the aim of identifying gaps and strengthening multisectoral coordination and collaboration.

It began by conducting a literature review and active search to identify the available preparedness and response plans developed or revised in Tanzania from 2006 to 2015. It also reached out to government officials and partner organizations through emails and telephone calls.

Five Preparedness and Response Plans (PRPs) were identified. These include the National Avian Influenza: Emergency Preparedness and Response Strategic Plan (NAI EPRP) 2006–2009; the Emergency Measures for Control of Rift Valley Fever in Tanzania (RVF CP) 2007; the National Avian and Pandemic Influenza Emergency Preparedness and Response Plan (NAPIP) 2011; the Tanzania Emergency Preparedness and Response Plan (TEPRP), 2012; and the National Ebola and Marburg Preparedness and Response Contingency Plan (NEM PRP), 2015. Content analysis of the plans was done using a checklist developed by the P&R Project to assess One Health inclusivity of the PRPs the results of which are summarised in Table 9 below.

**Table 9:** National preparedness and response plans (PRPs)

PRPs	Coordination	Sector involved in the plan	Context and purpose of the plan
1. Tanzania emergency preparedness and response plan (TEPRP), 2012	Prime Minister’s Office (PMO)	Multiple sectors and wide distribution of primary responsibilities among sectors including non-state actors, volunteer organizations, etc.	A multi-hazard functional plan that facilitates coordination to address any emergency, major disaster or imminent threats that Tanzania may face
2. National Avian and Pandemic Influenza Emergency Preparedness and Response Plan (NAPIP) 2011	Prime Minister’s Office	Ministry of Livestock Development and Fisheries (MLDF), Ministry of Natural Resources and Tourism (MNRT), Ministry of Health and Social Welfare (MOH & SW) as the lead ministries	Revision of a previous national avian influenza PRP of 2006–2009 to prevent incursion and spread of both the avian and human pandemic influenzas, taking into account new knowledge and dynamics in the influenzas
3. National Avian Influenza: Emergency Preparedness and Response Strategic Plan (2006/2007–2008/2009) (NAI EPRP)	Prime Minister’s Office	Multiple sectors with Agriculture, Health and Environment stated as lead sectors in most aspects of the plan	Developed in response to imminent threats of spread of highly pathogenic avian influenza (HPAI) from Asia. It followed multisectoral guidelines developed by FAO, OIE and WHO. Its purpose was to prevent incursion and spread HPAI in Tanzania
4. National Ebola and Marburg Preparedness and Response Contingency Plan, 2015 (NEM PRP)	PMO with Ministry of Health and Social Welfare as lead sector	Multiple sectors involved with Health Sector stated as lead sector in most aspects of the plan	Developed in response to imminent threat of spread of Ebola following the 2014 epidemic in some countries in West Africa. Guided by WHO guidelines to prevent its incursion and spread
5. Emergency Measures for Control of Rift Valley Fever in Tanzania, 2007 (RVF CP)	PMO	Ministry of Livestock Development and Fisheries (MLDF), Ministry of Natural Resources and Tourism (MNRT), Ministry of Health and Social Welfare (MOH & SW) as the lead ministries	Developed during a Rift Valley Fever outbreak in Tanzania as an emergency plan to control the outbreak

#### Strengths and gaps of the Tanzanian PRPs

Anchoring coordination of the PRPs in the PMO was the major strength. Three of the plans are firmly coordinated by the PMO under the Disaster Management Committee (DMC) and Tanzania Disaster Relief Committee (TANDREC), both of which are multisectoral. These are the National Avian and Pandemic Influenza Emergency Preparedness and Response Plan (NAPIP) 2011; Tanzania Emergency Preparedness and Response Plan (TEPRP), 2012; and National Avian Influenza: Emergency Preparedness and Response Strategic Plan (NAI EPRP) (2006/2007–2008/2009)**.** They all operate under the stewardship of a steering committee of permanent secretaries.

The main weakness was that two of the PRPs were still operating under respective sector ministries. For example, although the institutional framework for operationalizing the national Ebola and Marburg Preparedness and Response Contingency Plan (NEM PRP, 2015) is at the PMO office, in practice the involvement of the animal health sector and environment are not elaborated in the operational plan and have no financial allocations. The role of veterinary authorities in containment of the diseases in animals is missing in the plan. The Emergency Measures for Control of Rift Valley Fever in Tanzania, 2007 (RVF CP) has similar deficiencies in that reduction of human infection and human case management are stated but not elaborated as activities in the document. The role of the environment sector is also not supported by activities.

Following this review, the Tanzanian One Health platform is enabled to coordinate stakeholders to make changes addressing the identified gaps. This will result in more robust multisectoral preparedness and response plans contributing to improving Tanzania’s scores on the WHO Joint External Evaluation (JEE) [10] and OIE standards for veterinary services (OIE-PVS) [11] regarding preparedness and response to public health threats.

It is noteworthy that when conducting the JEE and OIE assessments, Uganda and Tanzania called for synchronization of the exercises so that they are conducted jointly and not separately “in silos” as has been the case so far, because they involve the same stakeholders for the same purpose.

### Other One Health interventions supported by the NOHP

One of the P&R Project’s key learnings has been that once National One Health Platforms are created and institutionalized appropriately, such as integrated into the national health security structure, they can serve a variety of multi-sectoral purposes that go beyond zoonotic diseases. These include supporting human and animal diseases surveillance systems, national antimicrobial resistance (AMR) programs, and addressing endemic diseases [12, 13]. The Tanzanian OHCD is imbedded within the PMO DMD as an integral part of the national health security structure [12].

In 2017 Tanzania became the first country to develop a National Action Plan for Health Security (NAPHS), initiated by the IHR team to address gaps identified by the Tanzanian JEE of February 2016 [12]. It involved nationwide participatory consultations under the coordination of the Prime Minister’s Office delegated to the National One Health Coordination Unit and overseen by the Inter-ministerial committee. The Ministry of Health, Community Development, Gender, Elderly and Children led the exercise. Participants included OH ministries listed in Table 1 and other key stakeholders from academia, research institutions and One Health networks. A series of planning workshops with key ministries, allied institutions, and implementing partners were conducted leading to the identification of priority activities to implement the JEE recommendations and fill the identified gaps. The planned activities were costed and approved as the NAPHS.

Given the need to maximize buy-in across sectors and deepen relationships forged during the JEE process, stakeholders agreed that the One Health Coordination Desk and inter-ministerial steering committee in the Prime Minister’s Office should be empowered and strengthened as the entity responsible for the plan’s implementation. Thus, the role and value of the One Health platform were further extended and validated.

## Conclusion

This case study has provided a “deep dive” into the development of the national One Health platform in Tanzania highlighting the facilitative role of regional networks, research and academia in championing the process. It illustrates the time and the commitment required of multiple partners to build a functional multi-sectoral coordination mechanism, and different challenges that may be encountered along the way [14, 15]. It also illustrates the critical role of political commitment and national ownership and leadership, emphasizing the importance of engaging policy and decision makers right from the beginning and continuously to ensure that knowledge and evidence remain not only with the technical experts, but are readily translated into policy and practice to benefit populations. It further confirms that the five dimensions identified in the Background section of this paper and by which it is organized are a good guide for establishing the necessary basic capacities for a functional MCM and allows it to grow and build strength while implementing OH activities. The Tanzania OH platform already demonstrates some of the expected benefits of a functioning MCM [16].

## Data Availability

All data and material for this publication is stored by Development Alternatives INC, which was the implementing agency for the project and may be made available under request to the director DAI Global Health through the corresponding author.

## Abbreviations

AAR: After Action Review
AFENET: African Field Epidemiology Network
AMR: Antimicrobial resistance
CWGESA: Cysticercosis Working Group in East and Southern Africa
DMD: Disaster Management Department
GARP-TZ: Global Anti-Microbial Resistance Partnership-Tanzania
MCM: Multisectoral coordination mechanism
MoU: Memorandum of understanding
MP: Member of Parliament
NAIEPRP: National Avian Influenza: Emergency Preparedness and Response Strategic Plan
NGOs: Non-Governmental Organizations
NOHP: National One Health Platform
NOHRMS: National One Health Resource Mobilization Strategy
NOHSC: National One Health Steering Committee
NOHSP: National One Health Strategic Plan
OH: One Health
OHCD: One Health Coordination Desk
OHCEA: One Health Central and East Africa
OHCU: One Health Coordinating Unit
P&R: Preparedness and Response
PMO: Prime Minister’s Office
PO-RALG: President’s Office - Regional Administration and Local Governments
PRP: Preparedness and Response plan
R&D: Research and Development
RVF: Rift Valley Fever
SACIDS: Southern African Centre for Infectious Disease Surveillance
TAC: Training Advocacy and Communication
TVLA: Tanzania Veterinary Laboratory Agency
TWGs: Technical Working Groups
USAID: United States Agency for International Development
